# Anti-parasitic activity of garlic (*Allium sativum*) and onion (*Allium cepa*) extracts against *Dactylogyrus spp.* (Monogenean) in Nile tilapia *(Oreochromis niloticus)*: Hematology, immune response, histopathological investigation, and inflammatory cytokine genes of gills

**DOI:** 10.1186/s12917-024-04187-5

**Published:** 2024-07-26

**Authors:** Rasha Reda, Alshimaa A. Khalil, Mohamed Elhady, Safaa I. Tayel, Enas A. Ramadan

**Affiliations:** 1 Department of Aquatic Animal Medicine, Faculty of Veterinary Medicine, Zagazig University, Zagazig, 44511 Egypt; 2 National Institute of Oceanography and Fisheries (NIOF), Al Qanater Al Khairia, 13723 Egypt

**Keywords:** *A. Sativum* extract, *A. Cepa* extract, Nile tilapia, *Dactylogyrus spp.*, Immunological variables, Cytokine genes, Histopathology

## Abstract

**Background:**

Gills monogenean infestation causes significant mortalities in cultured fishes as a result of respiratory manifestation. Medicinal plants are currently being heavily emphasized in aquaculture due to their great nutritional, therapeutic, antimicrobial activities, and financial value.

**Methods:**

The current study is designed to assess the effect of garlic *(Allium sativum)* and onion *(Allium cepa)* extracts as a water treatment on the hematological profile, innate immunity, and immune cytokines expression besides histopathological features of gills of Nile tilapia (*Oreochromis niloticus* L.) infected with gills monogenetic trematodes (*Dactylogyrus sp*.). Firstly, the 96-hour lethal concentration 50 (96 h-LC_50_) of garlic extract (GE) and onion extract (OE) were estimated to be 0.4 g/ L and 3.54 g/ L for GE and OE, respectively. Moreover, the in-*vitro* anti-parasitic potential for (GE) was found between 0.02 and 0.18 mg/mL and 0.4 to 1.8 mg/mL for OE. For the therapeutic trial, fish (*n* = 120; body weight: 40–60 g) were randomly distributed into four groups in triplicates (30 fish/group, 10 fish/replicate) for 3 days. Group1 (G1) was not infected or treated and served as control. G2 was infected with *Dactylogyrus spp*. and not exposed to any treatment. G3, G4 were infected with *Dactylogyrus sp*. and treated with 1/_10_ and 1/_5_ of 96 h LC_50_ of OE, respectively. G5, G6 were infected with *Dactylogyrus sp*. and treated with 1/_10_ and 1/_5_ of 96 h LC_50_ of GE, respectively.

**Results:**

No apparent signs or behaviors were noted in the control group. *Dactylogyrus spp.* infected group suffered from clinical signs as Pale color and damaged tissue. *Dactylogyrus spp.* infection induced lowering of the hematological (HB, MCH, MCHC and WBCs), and immunological variables (lysozyme, nitric oxide, serum Anti- protease activities, and complement 3). the expression of cytokine genes *IL-ß* and *TNF-α* were modulated and improved by treatment with *A. sativum* and *A. cepa* extracts. The obtained histopathological alterations of the gills of fish infected with (*Dactylogyrus spp*.) were hyperplasia leading to fusion of the gill filament, lifting of epithelial tissue, aneurism and edema. The results indecated that G4 and G5 is more regenarated epithelium in compare with the control group.

**Conclusion:**

*A. sativum* and *A. cepa* extracts enhance the blood profile and nonspecific immune parameters, and down-regulated the expression level of (*IL-1β* and *TNF-α*).

## Background

In the last decades, the aquaculture practices have increased worldwide. The Nile tilapia (*Oreochromis niloticus*) ranks the third among all fish species in terms of production volume [1]. This large production is followed by the necessity for larger spaces in fish farms for rearing the fish properly. However, instead of increasing space, high stocking density is usually carried out by farmers [2]. This condition impacts the fish’s quality of life and welfare by altering food competition and consumption, growth, and the potential for non-adaptive stress that consequently generating the diseases including the parasitic infection such as monogenetic trematodes [3].

The consequences of monogeneans’ epizootics and the challenges associated with treating them in rearing units make them a challenge for the production of fish [4]. High temperatures, high fish densities, and bad water quality are favorable to monogenean parasites and their transmission dynamics [5]. The hazardous effect of monogenea that the parasite has direct and simple life cycle as *Cichlidogyrus* eggs hatch in 2 to 6 days at temperatures between 20 and 28 °C, releasing free-swimming miracidia (infection phase). Within 4–6 days, they reach maturity and attach to their hosts, where they can spend up to 40 days [6]. These parasites take advantage of many stressors that influence their hosts while farming intensively [7, 8]. Additionally, through primary or secondary infections, their presence can cause fish mortality and severe economic losses [9]. Dactylogyrus *spp.* (Monogenean) could be considered as one of the most prevalent diseases affecting skin and gills, which included irritation, severe destruction of the gills, impaired breathing as well as severe losses too [10].

Strategies for parasitic eradication in aquaculture have been disregarded for a long time. The majority of research has been devoted to finding ways to lessen the financial losses brought on by parasitic diseases in farmed fish, and the practical solutions are mostly based on short-term chemical treatments that aim to directly eliminate the parasites or restrict transmission [11].

The most frequently used treatments for monogenetic trematodes in aquaculture included a number of chemical substances such as formalin, Hydrogen peroxide, Potassium permanganate and Praziquantel. These toxic substances are bio-accumulated in fish tissue [12] and in human body through the food chain, which makes them hazardous to the environment and human health [13]. Thus, in recent years, natural plant products have been considered as a means of controlling parasites in aquaculture and removing issues brought on by the use of chemicals [14]. The effects of extracts, essential oils and bioactive metabolites from diverse terrestrial and aquatic plants on fish parasites have been studied as an alternative treatment against different parasitic species of fish [11, 14–20].

Among the herbal plants, Allium (family: Amaryllidaceae) is a monocot genus with 800 species that are found all over the world and have various physical traits [21]. According to [22], garlic *(Allium sativum* L.) is the most pertinent species in the genus and onion (*Allium cepa* L.), is the Allium species that is used most frequently to treat common ailments. Since ancient times, *A. sativum*, *A. cepa*, and other plants rich in bioactive chemicals have been employed for therapeutic purposes [14, 23, 24]. Numerous authors have investigated the effects of *A. sativum* on certain parasites [11, 25–30].

One of the most frequently mentioned effects of *A. sativum* consumption on fish is its anti-parasitic effects, which are mostly attained by immersing in water [31]. The effects of *A. sativum* as a dietary supplement and as an extract for immersion baths used in the growth of aquatic species, both in fish and crustacean production, have been examined in a number of researches. Additionally, study on specific fish species *like Cyprinus carpio Oreochromis niloticus*, and *Oncorhynchus mykiss* as well as crustaceans like the white leg shrimp is of particular interest [14].

In fact, organosulfur compounds presented in *A. cepa* have been shown to have therapeutic effects on cancers, worms, fungus, viruses, bacteria, and protozoa [32]. Allicin, which was first identified and characterized in *A. sativum*, is the primary sulfur component present in the genus Allium. When fresh *A. sativum* is minced or crushed, an enzyme reaction turns alliin into this molecule. The synthesized allicin is unstable and quickly transforms into a number of sulfur-containing compounds, including S-allyl cysteine, Diallyl Disulfide, and S-methyl cysteine [33].

The most popular variety of the genus Allium is *A. cepa*, generally known as the bulb onion or common onion. According to [33], freshly cut *A. cepa* frequently produce a volatile fluid like syn-propanethial-Soxide, which has a variety of biological activities including anti-parasitic efficacy. The current study investigated the possible use of in-*vivo* anti-parasitic activity of *A. sativum* and *A. cepa* extracts against Dactylogyrus *spp.* (Monogenean) recovered from Nile tilapia (*Oreochromis niloticus*) gills. The investigation was carried out by evaluating the immunological, blood parameters, histopathology, and cytokine gene expression of gills.

## Methods

### Preparation of *A. sativum* and *A. cepa* aqueous extract

The *A. sativum* and *A. cepa* were obtained from El-Kanaterl khairia market. The garlic and onion were ground and mixed in a kitchen blender, the mixture was then filtered with a strainer [34]. To prepare the stock solution for the in vitro experiment, 10 gm of ground onion were weighed and then added to 20 ml of cold water (0.5 g ml^− 1^). While the freshly generated extract concentrations for garlic and onions in vivo were determined using LC_50_ (1/_10_ and 1/_5_ of LC_50_) [34].

### Phytochemical screening of *A. sativum* and *A. cepa* aqueous extract

Phytochemical analysis of Phytochemical components, including saponins, tannins, flavonoids, alkaloids, saponins, phenols, and balsam, were detected in the Aqueous extracts of *A. sativum* and *A. cepa* using the method reported by [35–38].

In order to determine the amount of saponins, 0.5 g of plant extract and 5 mL of distilled water were combined in a test tube and thoroughly shaken. The continual foaming formed indicated the presence of saponins. Tannin determination was performed by combining 0.5 g of extracts with 10 mL of bromine water. In order to quantify the amount of flavonoids, two milliliters (2 mL) of 2.0% sodium hydroxide and 0.5 g of plant extract were mixed in a test tube. The decolorization of bromine water indicated the presence of tannins. The mixture’s golden coloring turned colorless at the addition of two drops of 10.0% hydrogen chloride, indicating the presence of flavonoids. Determination of phenol: Ten milligrams of the extract were combined with two drops of ferric chloride solution. The emergence of a blue-black coloration indicated the presence of phenol. To identify the alkaloids, 0.5 g of extract was dissolved in 2 mL of 2.0% sulfuric acid, and the mixture was then heated for duration of two minutes. Three drops of Dragendorf’s reagent were added to the filtrate after the mixture had been filtered. Alkaloids were present because an orange-red precipitate was produced.

### The in-vitro anti-parasitic assay the monogenean parasites

The in-*vitro* anti-parasitic assay the monogenean parasites were dislodged from the gills of infected fish using fine insect pins and transferred to small petri dishes. Any worms that died were discarded. Then, drop the freshly prepared GE (0.0, 0.02, 0.04, 0.06, 0.08, 0.1, 0.12, 0.14, 0.16, and 0.18 µg/mL) and OE (0.0, 0.2, 0.4, 0.6, 0.8, 1, 1.2, 1.4, 1.6, and 1.8 µg/mL) on these dishes. The motility and vitality of the parasite were observed immediately after application of the treatment under light microscope and 5 min later then till time of death were noted according to the method carried by [39].

### Fish and cultural conditions

For the in-*vivo* assay, infected Nile tilapia (*Oreochromis niloticus*) with gill monogenean, Dactylogyrus *spp.*; with average body weight range from 40 to 60 g were obtained from the National Institute of Fisheries and Oceanography, El-Kanater El khairia Fish Farm, Qalyubia, Egypt. The fish were stocked in 96-L glass aquaria containing de-chlorinated tap water, supplied with continuous aeration from a central air compressor. The fish were acclimated before the beginning of the experimental trial for two weeks and were fed on commercial diet (crude protein 30%). the diet was purchased (Aller Aqua Egypt company, Giza governorate Egypt). Siphoning of fish excreta carried-out daily for removal of the excreta and complete water exchange was done three times weekly. The water parameters were maintained according to the requirement of Nile tilapia before the start of the experiment, the fish health status was checked according to [40].

### Determination of the 96 h- LC_50_ of GE and OE and calculation of therapeutic dose

The 96 h- LC_50_ of GE and OE was determined using 180 fish. The fish were exposed to ten different concentrations of GE (5, 10, 15, 20, 25, 30, 35, 40, 45 mg L^− 1^) and OE (0, 50, 100, 150, 200, 250, 300, and 350 mg L^− 1^) for 96-h without feeding and water exchange. The fish mortalities were recorded and the 96 h- LC_50_ of GE and OE was determined according to [41]. The dose concentrations of both GE and OE that used for the in vivo treatment study was established to be equal to 1/_10_ and 1/_5_ of the 96-h LC_50_. *A. sativum* dose calculated1/_5_ of LC_50_ equal to (0.08 g L^− 1^) and 1/_10_ of LC_50_ equal to (0.04 gL^− 1^) While *Allium cepa* dose calculated 1/_5_ of LC_50_ equal to (0.7 g L^− 1^) and 1/_10_ of LC_50_ equal to (0.35 gL^− 1^).

### Experimental design for treatment assay

The fish were randomly assigned to six groups; each group has three replicates (*N* = 30 fish/group; 10 fish/replicate) for 3 days. The first group (G_1_) was fed on a basal diet, without being infected or and regarded as a control. The second group (G_2_) (monogenea spp) fish was infected with monogenean and not exposed to any treatment. The third group (G_3_) was infected with monogenean and treated with 1/_10_ of 96 h LC_50_ of freshly prepared onion. The fourth group (G_4_) was infected with monogenean and treated with 1/_5_ of 96 h LC_50_ of freshly prepared onion. The fifth group (G_5_) was infected with monogenean and treated with 1/_10_ of 96 h LC_50_ of freshly prepared garlic. The sixth group (G_6_) was infected with monogenean and treated with 1/_5_ of 96 h LC_50_ of freshly prepared garlic. The clinical signs, postmortem lesions, and the behavioral changes of the experimented fish were recorded daily.

### Sampling and analysis

After the 3rd day of the treatment, fifteen fish were randomly selected from each group (five fish/ replicate) and anesthetized using clove oil (Oleum, Egypt) within 3 min with a dose of 0.033 ml L^− 1^ [43]. Fish are putting in a container with dechlorinated water containing the anesthetic solution. For every replicate in every treatment, this process was repeated. When fish showed signs of decreased opercular pumping and pectoral fin thumping, they were seemed anesthetized. Blood samples were taken from the fish caudal peduncle using 3 ml syringe with heparin for determination of the hematological parameters. Other blood samples were collected in small sterile tubes for serum separation and left to coagulate for 15–20 min at 4 °C before centrifugation for 15 min at 3000 *x g* at room temperature to separate serum and stored at -20 °C until further biochemical analysis and immunological assay. Then, fish were sacrificed and samples of the gills (9 samples/ group) were collected for the genomic assay. Gills samples were also dissected for histopathological examination.

### Hematological parameters

Red blood cells (RBC) and white blood cells (WBC) were measured using an automated hematology analyzer (Hospitex Diagnostics, Sesto Fiorentino, Italy) as blood parameters. Following sampling, hemoglobin (Hb), packed cell volume (PCV), mean corpuscular volume (MCV), and mean corpuscular hemoglobin (MCH) were measured right away using techniques outlined by [42].

### Immunological assay

Anti-protease activity according to methods of [43] as cited by [44], 10 µL of plasma was incubated with 10 µL of a trypsin solution for 10 min at 22 °C in polystyrene micro tubes. To the incubation mixture, 100 µL of phosphate buffer and 125 mL of azocasein were added and incubated for 1 h at 22 °C. Finally, 250 µL of 10% trichloro acetic acid (TCA) were added to each micro tube and incubated for 30 min at 22 °C. The mixture was centrifuged at 10,000 *× g* for 5 min at room temperature. After that, 100 µL of the supernatant was transferred to a 96 well-plate containing 100 µL of 1 N NaOH per well. The optical density (OD) was read at 450 nm in a Synergy HT microplate reader. Phosphate buffer in place of plasma and mucus and trypsin served as blank, whereas the reference sample was phosphate buffer in place of plasma.

Complement 3 (C3) was determined using BioSoure Co. Eliza Kits Cat. No. MBS005953, Reagent 1 was blended slowly with distilled water (2 mL), standard liquid, and serum. The absorbance was estimated at 340 nm after a 5-min of incubation at 37 °C. After that the tubes were filled with Reagent 2 then the absorbance was measured. A C3 standard curve was created using the same process. The measurement of serum complement 3 (C3) following the producer’s guidelines. Lysozyme activity using Worthington Biochemical Corporation Kites and following the producer’s guidelines, the measurement of lysozyme activity and the ultraviolet inactivation of lysozyme was assayed by modification of the turbimetric procedure of [45]. Nitric oxide was measured according to methods used by [46]. The Griess reagent was applied to 100 mL of each serum sample in a micro titer plate and incubated at 27 °C for 10 min, then NO level was measured using a spectrophotometric as described by [47].

### Transcriptomic analysis

According to [48], Gills tissue samples was taken, cleaned with PBS buffer (pH 7.2), stored in an RNA later, and kept at -80^◦^ C until transcriptomic analysis was performed in the lab to check for the presence of IL-β1 and TNFα gene. mRNA expression of interleukin 1 beta (Il-1β) and tumor necrosis factor alpha (TNFα) in gill tissues was determined by RT-PCR. Te amplifcation of cDNA, a house hold-gene (Actin- β) mix has been included with SYBR Green; the primers used are illustrated in Table (1). Sequences for the cytokines Il-1β (NCBI: DQ061114**)** according to [49], TNFα (NCBI: AY428948) according to [50, 51] and Actin- β (NCBI: EU887951) according to [52].

**Table 1:** The primer used in the transcriptomic analysis

Gene coding	Primer Sequence(F)	Primer Sequence(*R*)	Annealing Temperature (°C)	ACCESSION	Referance	Gene size
IL-β1	TGC ACT GTC ACT GAC AGC CAA	ATG TTC AGG TGC ACT ATG CGG	62	DQ061114	[50]	306 bp
TNF-α	GCT GGA GGC CAA TAA AAT CA	CCT TCG TCA GTC TCC AGC TC	60	AY428948	[51, 52]	744 bp
Actin- β	TGG CAT CAC ACC TTC TAT AAC GA	TGG CAG GAG TGT TGA AGG TCT	60	EU887951	[53]	543 bp

### Histopathological investigation

Tissue specimens from gills were collected and dissected out from the control and tested groups, fixed at 10% neutral buffered formalin for 24 h, and then rinsed with water, dehydrated serially in ethyl alcohol, cleared by xylol, and rehydrated with decreased ethanol concentrations. The fixed tissues embedded in paraffin wax and sectioned at 3–5 microns, then stained with hematoxylin and eosin (H&E) according to [53] and then examined by light microscopy. To quantify the severity of the revealed major histopathological change, different levels of severity were defined according to the methods mentioned by [54]. The degree of damage to the gills is the sum of the multiplied importance factors of all changes found within the examined gill filament according to the method used by [55] and [56].

### Statistical analysis

The data normality was checked by the shaprio-wilk test. Afterward, the data were statistically investigated using a one-way analysis of variance (ANOVA) (SPSS version 16.0, SPSS Inc., Chicago, IL, USA).

## Results

### Phytochemical screening of the plants

The findings of the phytochemical screening of the GE and OE revealed the presence of significant phytochemicals in the raw forms of the plants, including saponins, alkaloids, tannins, flavonoids, cardiac glycosides, terpenoids, and resins. But neither balsam nor phenols were detected in the two plants’ unprocessed aqueous forms Table (2).

**Table 2:** Qualitative Phytochemical components of the raw form of garlic (*Allium sativum*) and onion (*A. cepa*)

	Aqueous garlic	Aqueous onion
Alkaloids	+	+
Flavonoids	+	+
Tannins	+	+
Saponins	+	+
Balsam	ND	ND
Cardiac glycosides	+	+
Terpenoids	+	+
Resin	+	+
Phenols	ND	ND

Key: + = present; - = absent

### In vitro anti-parasitic activity of plant extracts against Dactylogyrus *spp.*

The parasite in the untreated control group survived an average of 1–2 h while the range of survival time in the *GE* treated groups (4–5 min.) which show complete death of monogenean after 5 min. of exposure in the other hand, it causes damage to the gills tissue and changing its color to brown. OE treated groups showed complete death after 0.25 h the range of survival was (0.2–0.25) h (Table 3). According to the obtained results (*A. sativum* and *A. cepa*) was the chosen plant extracts for the immersion in vivo treatment. The 96-h LC_50_ of GE was found to be 0.4 g/L and it was found to be 3.45 g/L for OE (Tables 4 and 5).

**Table 3:** The in vitro effect of different plant extracts on *Dactylogyrus spp*

Plant extract	Concentration	Survival range (hr)
Untreated group	--	1–2
Garlic (*Allium sativum*)	0.5 g ml^− 1^	0.06–0.08
Onion (*Allium cepa*)	0.5 g ml^− 1^	0.2–0.25

**Table 4:** Actual estimation of 96 h mortalities LC_50_ in Nile tilapia fingerling exposed to different concentration of garlic (*Allium sativum*)

Group *N* = 8	Concentration mg/L	Number of dead fish at 96 h	a	b	axb
1	0	0	0	0	0
2	10	0	5	0	0
3	15	1	5	0.5	2.5
4	20	4	5	2.5	12.5
5	30	7	10	5.5	55
6	45	7	15	7	105
7	60	8	15	7.5	112.5
8	75	8	15	8	120

96hrsLC_50_ = highest dose- Σaxb/n

A = factors between two successive dose

B = the mean of dead fish in each group

Σaxb = sum of axb

n = Number of fish in each group

**Table 5:** Actual estimation of 96 h mortalities LC50 in Nile tilapia fingerling exposed to different concentration of onion (*A. cepa*) extract

Group *N* = 8	Concentration mg/L	Number of dead fish at 96 h	a	b	axb
1	0	0	0	0	0
2	50	0	25	0	0
3	100	0	50	0	0
4	150	2	50	1	50
5	200	3	50	2.5	125
6	250	5	50	4	200
7	300	8	50	6.5	325
8	350	8	50	8	400

96hrsLC_50_ = highest dose- Σaxb/n

A = factors between two successive dose

B = the mean of dead fish in each group

Σaxb = sum of axb

n = Number of fish in each group

### Antiparasitic efficacy

The anti- parasitic efficacy of garlic and onion revealed that the monogenean parasite found in gills of Nile tilapia were significantly decreased by garlic and onion exposure as G_4_, G_5_ and G_6_ (85.7%,90.5%,100%) respectively show the most effectiveness on parasite in G_6_ (Table 6).

**Table 6:** Effect of *A. Sativum* and *A. cepa* extracts on clinical signs and postmortem changes of survival infected Nile tilapia after 72 h

Clinical signs	Experimental group					
	G1	G2	G3	G4	G5	G6
NO. of survived fish	23/24	15/24	17/24	21/24	21/24	18/24
Low food intake	0/24	9/24	3/24	2/24	1/24	2/24
Loss reflex	--	2/24	--	--	--	--
External skin lesion	--	7/24 Pale coloration, detached scale and damaged fins	--	--	--	3/24 Slight damage in fin edges
Postmortem changes	--	13/24 Gills was Pale color and damaged	--	--	--	6/24 Slight damage to gill
Reduction of infection on the gills (%)	95%	0%	64.7%	85.7%	90.5%	100%

### Hematological profile

As shown in (Table 7), the lowest values (*P <* 0.05) of RBCs, WBCs, Hb, PCV (%), were seen in infested and not treated group *Dactylogyrus spp* (G2) compared with the control group. *Dactylogyrus spp +* GE *group* induced significant improvement (*P <* 0.05) in these variables followed by *Dactylogyrus spp +* OE group when compared with the *Dactylogyrus spp* group.

**Table 7:** Hematological profile of Nile tilapia experimentally infected with *Dactylogyrus spp.* and treated with *A. Sativum* and *A. cepa* extracts for 72 h

		platelet	RBCs	Hct	HB	MCV	MCH	MCHC	WBCs	Lymphocyte	Neutro Staff	Neutro Seg.	Eosinophil	Monocyte
G1	109.000 b		0.930 c	19.650 b	9.745 a	158.250 a	65.750 a	36.700 a	17.330 a	21.000 a	2.000 b	46.000 a	4.000 a	3.000 c
G2	186.400 ab		3.340 a	26.340 ab	7.435 ab	11.220 f	20.755 c	22.670 a	45.510 a	29.500 a	4.500 a	54.500 a	5.500 a	4.000 abc
G3	148.390 ab		3.260 a	28.160 a	8.215 ab	115.215 b	31.765 b	26.495 a	57.185 a	25.000 a	2.500 b	59.000 a	5.500 a	5.500 a
G4	330.000 a		2.905 ab	23.700 ab	6.900 ab	103.600 c	28.350 bc	23.390 a	46.575 a	23.000 a	2.500 b	62.500 a	6.000 a	5.000 ab
G5	158.125 ab		3.290 a	25.600 ab	7.845 ab	95.645 d	35.060 b	33.675 a	49.610 a	28.000 a	2.000 b	61.000 a	5.000 a	3.500 bc
G6	203.500 ab		2.615 b	21.800 ab	5.900 b	86.750 e	25.700 bc	28.555 a	49.605 a	32.500 a	3.000 b	53.500 a	4.500 a	5.500 a
Pr > F(Model)		0.162	0.000	0.238	0.121	< 0.0001	0.000	0.376	0.390	0.693	0.017	0.305	0.416	0.063
Significant		No	Yes	No	No	Yes	Yes	No	No	No	Yes	No	No	No

a–f: Different letters in the same column indicate statistically significant differences (*P* < 0.05, Duncan’s test)

Pr > F: The *P*-value is associated with the F-statistic of a given effect and test statistic

### Immunological variables

The results showed significant reduction (*P <* 0.05) in the immune response (LYZ, NO, SAP and C3) of the *Dactylogyrus spp* group compared to the control group. Substantial improvement (*P <* 0.05) in these variables was noticed in the Dactylogyrus *spp + GE* group followed by the *Dactylogyrus spp +* OE group when compared to the *Dactylogyrus spp* group (Table 8).

**Table 8:** Effect of *A. Sativum* and *A. cepa* extracts on non-specific immune parameters of survival infected Nile tilapia after 72 h

	Lysozyme activity	Anti-protease activity	serum complement-3	Nitric oxide
G1	28.000 a	0.335 ab	117.000 a	0.039 a
G2	25.500 a	0.310 b	122.000 a	0.038 a
G3	29.500 a	0.375 a	108.000 a	0.033 ab
G4	28.000 a	0.380 a	108.000 a	0.033 ab
G5	27.500 a	0.380 a	118.000 a	0.031 b
G6	27.000 a	0.345 ab	112.500 a	0.039 a
Pr > F(Model)	0.910	0.132	0.300	0.061
Significant	No	No	No	No

a–b: Different letters in the same column indicate statistically significant differences (*P* < 0.05, Duncan’s test)

Pr > F: The *P*-value is associated with the F-statistic of a given effect and test statistic

LYZ: Serum lysozyme activity- SAP: Serum anti -proteases - C: Alternative complement – NO: Nitric oxide

### Cytokine gene expression

**Fig. 1:**
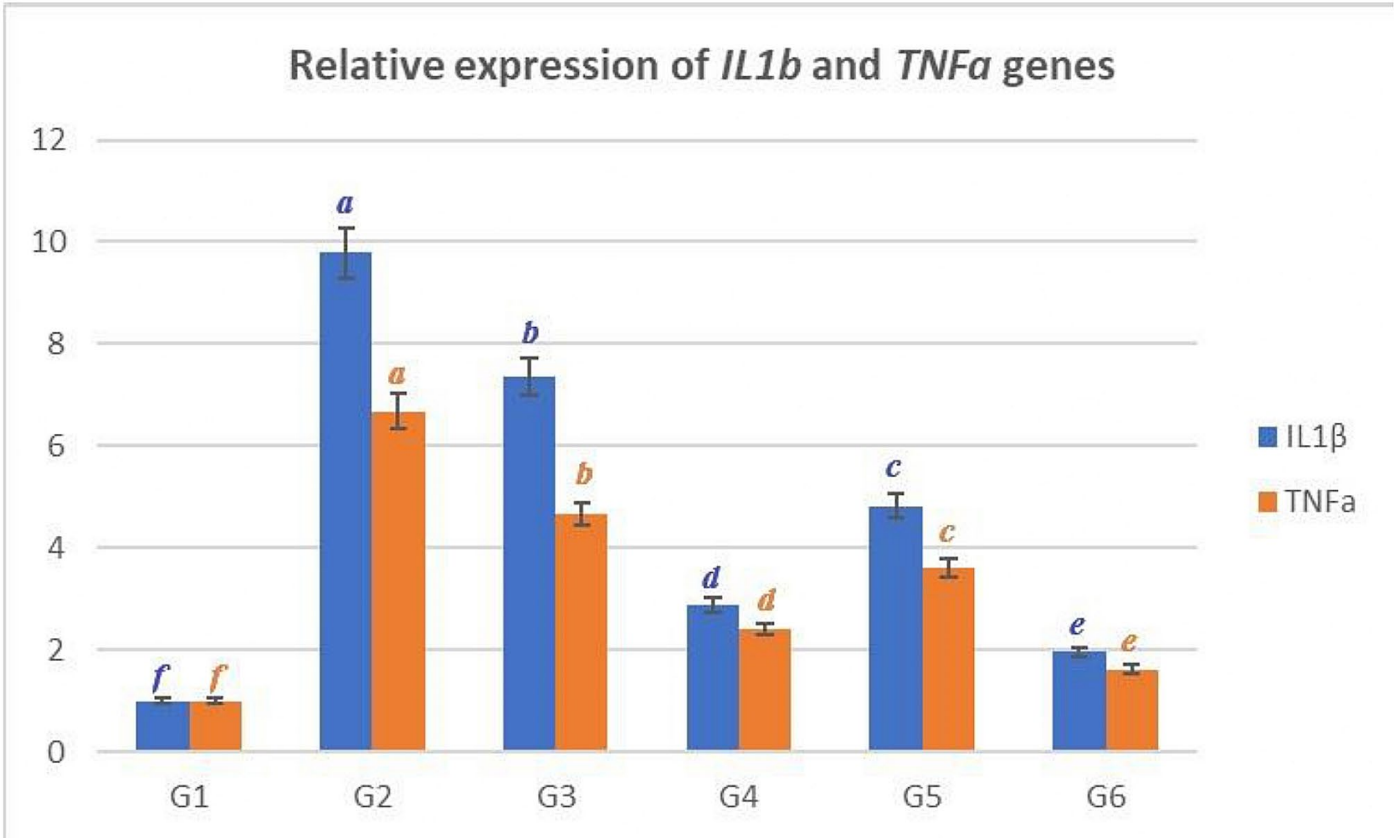
Graphical presentation of real-time quantitative PCR analysis of the expression of *IL-1β* and *TNFα* genes. show that G2 had significantly higher levels of *IL-1β* expression (9.78a fold) and *TNFα* expression (6.68 fold) compared to group G1 (1.00 f fold). Group G2 also had higher levels of *TNFα*expression (4.66 fold) than the control group G1. The *Dactylogyrus spp* + G6 group had significantly lower expression levels of these cytokines (*IL-1β* and *TNFα*) (1.96 e and 1.62 e), followed by G4 (2.87 d and 2.41 d) and G5 (4.82 c and 3.61 c)

Figure (1) illustrates that group G2 had significantly higher levels of IL-1β expression (9.78a fold) and TNFa expression (6.68 fold) compared to group G1 (1.00 f fold). Group G2 also had higher levels of TNFa expression (4.66 fold) than the control group G1. The Dactylogyrus spp + G6 group had significantly lower expression levels of these cytokines (IL-1β and TNFa), followed by G4, and G5, in that order.

### Histopathological findings of gills

Microscopic examination of examined Nile tilapia gills demonstrated alterations in the gill structure, the most pronounced alterations detected were hyperplasia, epithelial lifting, vasodilation, aneurism and edema, lymphocyte infiltration was also observed as indication of mild inflammation. the severity of this alterations is calculated by estimation of level of altration severity and if this altrations were reversible lesion in which exposure to irritants ends and the stressor is neutralized, or irreversible which lead to partial or total loss of the gill function.

**Fig. 2:**
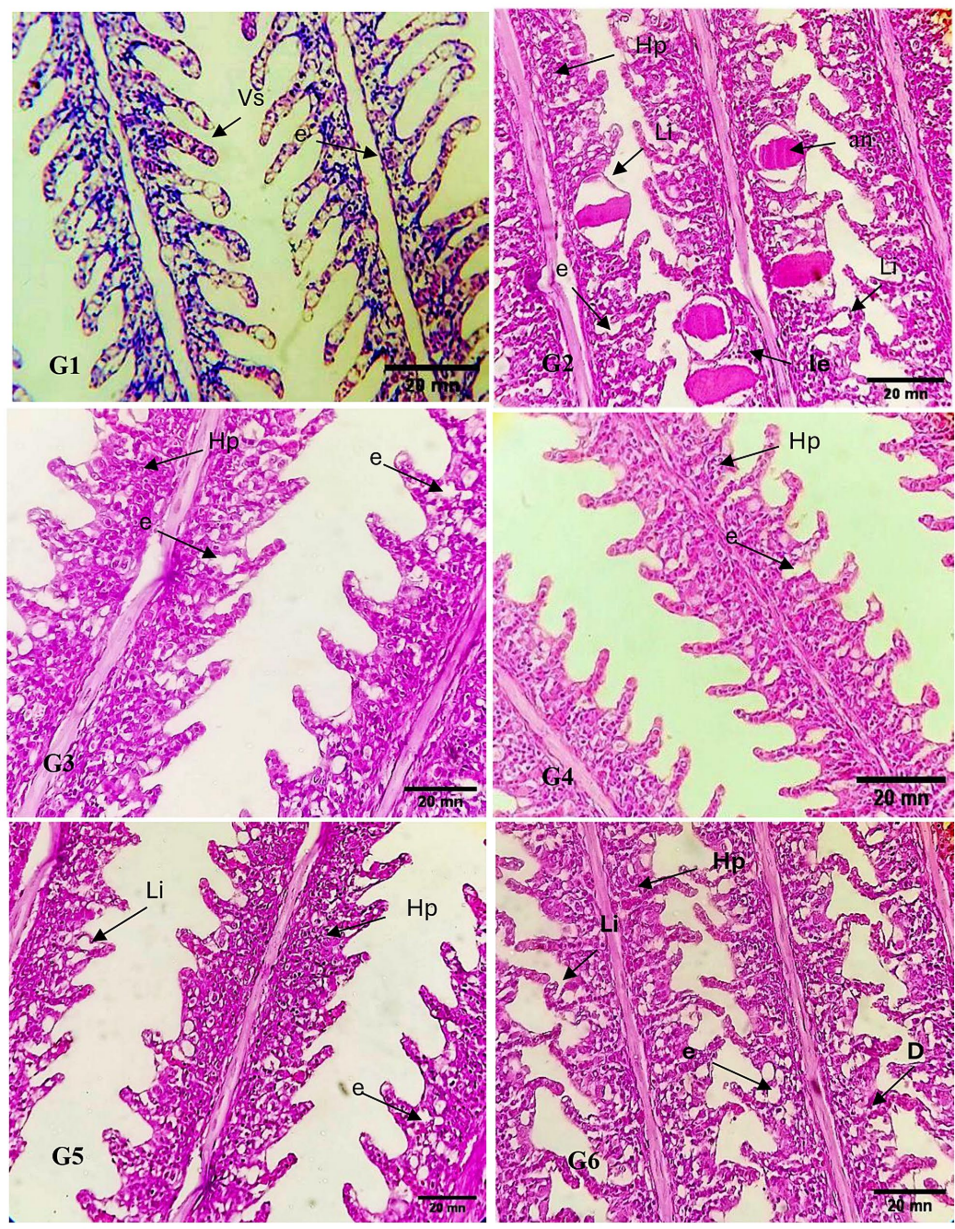
Sagittal section of *O. niloticus* gills revealed that control (G1): control group show slight vasodilation (vs) and edema(e). (G2) show Hyperplasia(Hp), lifting(Li) of epithelial tissue, aneurism(an), edema(e) and leukocyte infiltration(le). (G3) show Hyperplasia (Hp) and edema(e). (G4) show Hyperplasia (Hp) and edema(e). (G5) show Hyperplasia(Hp), lifting(Li) of epithelial tissue and edema(e). (G6) show Hyperplasia(Hp), lifting(Li), degeneration (D) of epithelial tissue and edema(e)

The control (G_1_) appeared nearly normal gills which is firm, while show slight vasodilation and edema. The *Datylogyrus spp* group(G_2_) showed show Hyperplasia(Hp), lifting(Li) of epithelial tissue, aneurism(an), edema(e) and leukocyte infiltration(le). *Dactylogrus spp* + OE group(G_3_) showed hyperplasia (Hp) inbetween the epithilial cells of the secondary lamellae and edema (level 2) which mean that the edema was deep within the filament epithelium, While *Dactylogrus spp* + OE (G_4_) show hyperplesia only at the base of secondary lamellae and edema (level 1) which mean that the edema was only occurring around the base of the lamellae. The *Datylogurus spp* + GE group(G_5_) showed hyperplasia (Hp) of seconary lamellae which stoped at the base of the filaments and oedema of the filament epithelium (level 1) which was only occurring around the base of the lamellae’s. The *Datylogurus spp* + GE group (G_6_) show degenaration at the base of secondary lamellae. This result indecated that (G_4_ and G_5_) is more regenarated epithilium in compare with the control group.(Fig. 2).

## Discussion

The ability of medicinal plants to enhance fish health and immunity, improving the host’s defense against infectious diseases, has recently piqued the interest of numerous researchers. In this context, the current study examined the potential addition of GE and OE to fish water (bath treatment) and its effects on the blood profile, innate immunity, and resistance of Nile tilapia to the infection with gill monogenea, *Dactylogyrus spp*.

Numerous herbal plants have beneficial benefits that counteract the side effects of synthetic chemical medications in the treatment of disease agents [16, 20, 27, 28, 30]. Studies on the treatment effects of OE on fish parasites are rather rare [11], in contrast to the studies carried on the treatment effects of GE on several Monogeneans in diverse fish species [28, 39]. The data on the anti-parasitic properties of GE and OE against *Dactylogyrus* species (gill monogenea) are presented in this study.

Concerning with Phytochemical screening of the extracts of *Allium sativum* and *Allium cepa* showed the presence important phytochemicals such as saponins, alkaloids, tannins, flavonoids, cardiac glycosides, terpenoids, and resins, this result was nearly similar to result described by [57] and [58] who revealed the presence of saponins, tannins, flavonoids in both aqueous *Allium sativum* and *Allium cepa* extract and absence of alkaloids in only aqueous *Allium sativum*. However, in our result balsam and phenols were not found in the raw aqueous forms of the both plants which come in line with [36] and [58].

Considering of the GE and OE that exerts anti-parasitic effect, the most effective concentration for GE was 0.5 g ml^− 1^ at 4–5 min while, OE 0.5 g ml^− 1^ for 20-25-minute revealing that GE was more effective against *Dactylogyrus spp.* than OE. In our study, the survival time of *Dactylogyrus spp* ranged from 5 min to 25 min in *GE* and *OE*-treated parasites. The results of the survival time ranges are consistent with those found in [39]. However, according to [59], the survival duration of *Neobenedenia sp.* (Monogenea) exposed to OE (dilution 1:10) and GE (dilution 1:10) was 8 h and 7.6 h, respectively. *A. sativum* and *A. sativum* -based products have been found to have anti-parasitic action for the ciliate *Cryptocaryon irritans* in *vitro*; however, in *vivo* testing using the guppy *Poecilia reticulata* infected with *C. irritans* failed to eradicate *C. irritans* [29]. *Neobenedenia sp.* in barramundi, *Lates calcarifer*, was reduced when *GE* was added to the meal [26]. Abd El-Galil [60] stated that Trichodinosis and Gyrodactylosis were completely eradicated from Nile tilapia at a ratio of 68% after exposure to *GE*. According to [39], Chinese freeze-dried *GE* had a 99% efficacy after just one treatment, and GE in the minced and granule forms reduced *G. turnbulli* by 66 and 75% after 3 more treatments.

*GE and OE’s* primary active components have been shown to have anti-parasitic activities for a variety of parasite species [32] depending on the quantity and duration. In this study, GE *and* OE showed an in-*vitro* anthelmintic activity against *Dactylog*yrus *spp*. (Monogenean) from the gills of Nile tilapia.

The anti-parasitic effects of GE and OE against *Dactylogyrus* spp could attributed to that GE and OE create polar and apolar bioactive compounds with pharmacological effects include cytotoxicity, antispasmodic, and anti-parasitic characteristics [24]. Repeated immersions could be required to boost the effectiveness of the therapy and enhance the likelihood that the parasite will come into touch with the powerful agents *of* GE or OE. The effectiveness of the immersions would not be high unless the plant’s primary active chemicals, like Allicin, could trigger apoptosis when they reached the parasite or damage the parasite’s vital enzyme system [61].

The findings of the present study demonstrated that various health indicators in Nile tilapia might be successfully improved by water containing GE and OE in addition to combating *Dactylogyrus spp*. The best results were found in the group of fish treated with the GE followed by the OE treated group as indicated by significant enhancement of in all blood indices. The increased production of leucocytes, red blood cells, and other blood components in the hematopoietic tissue, including the liver, may be responsible for the elevated hematological parameters in fish blood. This finding suggests that including GE in the water may boost immunity and aid in the combating the infection. The results may be attributed to that the Numerous phytochemicals, including those containing sulfur, are present in *A. sativum*, including ajoenes, thiosulfinates, vinilditine, sulfides, diallyl trisulfide, and cysteine [62]. Additionally, S-propylcysteine-sulfoxide and S-methyl cysteine-sulfoxide are found in GE. Depending on the temperature and moisture content, these compounds can form more than 50 metabolites [63] Alliin, which turns into allicin, N-acetylcysteine, S-allylcysteine, and S-allyl-mercapto cysteine are the secondary metabolites derived from the cysteine accumulated in plants of the Allium genus [63, 64]. Antioxidant, anti-inflammatory, and anti-cancer capabilities are all possessed by these active ingredients [65].

Since it is well known that the liver plays a significant role in fish hematopoiesis and that GE does not interfere with the activity of the enzymes alanine transaminase and aspartate aminotransferase, these findings can be attributed to the plant, which may cause stabilized cell membrane and protect the liver against harmful substances and free radical-mediated toxic damages to the liver cells. The decrease in liver enzymes is evidence of this. By boosting the potential for cell regeneration, *GE* aids the liver in maintaining normal function [66].

Fish have a higher reliance on innate immunity than mammals do for protection [67]. Through a process known as phagocytosis, the phagocytic cells (neutrophils and macrophages), which are a vital component of innate immunity, play a critical role in the elimination of infections. In order to increase their ability to phagocytose pathogens and kill them, macrophages also release powerful reactive oxygen known as NO [68]. In the current study, GE followed by OE as water extracts are significantly enhanced the non-specific immunological defenses (LYZ, NO, SAP and C3) in Nile tilapia as compared with that in the infected non-treated group. This may be due to potential biological and pharmaceutical effects including antioxidant, antibacterial and antiviral impacts. Allicin is an unstable, volatile, and cytotoxic liposoluble organosulfur compound [69]. With antiseptic, antiviral, antifungal, anti-parasitic, and antibacterial effects, it is also GE*’s* most significant active ingredient [70]. Allicin and ajoene, which are active ingredients used in veterinary medicine and livestock production [71, 72] have also been shown to have positive effects and anti-parasitic qualities on the zoo technical performance of farm animals [73, 74]. Several thiosulfinates, including allicin, are thought to be responsible for GE*’s* therapeutic effects in the treatment of monogenetic infection brought on by *Dactylogyrus* spp [75, 76]. Pathways involving interferon-gamma, tumor necrosis factor, interleukin (IL-12P70), and T-cells, enhance the host immunological response [69]. Moreover, organosulfur, polysaccharide, and fructan substances are responsible for *GE*’s immune stimulatory affects [77] a rise in the number of lymphocytes in the blood. Due to the fact that *GE*’s active ingredients encourage the growth of defense cells and that an increase in lymphocyte concentration indicates an increase in the inflammatory response, cell-mediated immunity, and/or humoral immunity, this has been seen [78].

Concerning the effect of *GE* and *OE* on the gene expression of Nile tilapia, Cytokines genes are the principal regulators of fish immunity associated with inflammation [79]. *IL-1β* and *TNF-α* are cytokines implicated in inflammatory response induction in fish [80]. The existence of those cytokines results in inflammatory responses [81]. In this study the expression level of *IL-1β* and *TNF-α* were considerably higher in *Dactylogyrus spp* group. The expression level of these cytokines were decreased in *Datylogrus spp* + GE followed by *Dactylogyrus spp +* OE group. These results may indicate that the 72 h bath treatment of Nile tilapia with freshly prepared extract of GE and OE down-regulated the expression level of (*IL-1β* and *TNF-α*). Therefore, the down-regulation of these genes observed indicate the positive effect of GE and OE in the inflammation. Marefati et al. [82] revealed that the Immunomodulatory effects of the *Allium cepa* and its derivative (quercetin) was shown by reduction cytokines as IL-1β and TNF-α. In addition, Kim et al. [83] revealed that the extract from whole onion (*Allium cepa L*.) significantly reduced the production of IL-1β and TNFα and their mRNA expressions indicating that whole onions had antioxidant and anti-inflammatory effects. Elgendy et al. [84] revealed that Dietary supplementation with *A. cepa* (0.5% *A. cepa* extract) reduced cadmium accumulation in fish organs and up-regulated *IL-1β* and *IFN*ɣ levels.

The fish gills are a multifunctional organ involved in respiration and homeostatic activities such as osmoregulation, metabolism, and circulation of hormones, nitrogen excretion, ion regulation and acid base balance [85]. Gills are among the most delicate structures of the fish body which have an external location so they are subjected to damage by pathogen (parasites, bacteria, fungi and virus) and/or toxicants (heavy metals, pesticide, drugs) [53]. When gill lamellae are exposed to pathogens and/or poisons, their epithelial cells increase in number (hyperplasia) as a defense mechanism, which reduces their surface area and delays the organ’s ability to breathe [86].

The obtained results revealed that the gill lamellae epithelial cell of Nile tilapia that had been infected with monogenea (*Dactylogyrus spp*.) are increased in (hyperplasia) leading to fusion of the gill filament, lifting of epithelial tissue, aneurism, edema and leukocyte infiltration nearly to results revealed by [87, 88]. These lesions may lessen the space for gas exchange, and even result in secondary infections that could result in significant illness with unfavorable effects [89–92]. Many parasites can induce histopathological alterations such as hyperplasia, aneurism, inflammation or even necrosis [93] those alterations are considered to be irreversible [56]. When we treated the experimented fish by adding GE and OE we obtained nearly improved gills, our finding agrees with Agbebi et al. [94] who indicated that there were no histological changes in African catfish; *Clarias gariepinus* fed garlic-containing diets. It should be noticed that there are limited scientific literature on the effect of onion and garlic supplementation on histological examination of fish gills as recorded by [95]. Therefore, we recommend adding *GE* and *OE* to fish water before infection or adding them to parents to protect the offspring and young fish.

## Conclusion

The present study indicated that watery addition of GE and OE can augment general health condition of Nile tilapia infected with gill monogenea by enhancing the blood profile and nonspecific immune parameters of the fish and improving the histological architecture of gills. The immersion treatment using the GE and OE had no adverse effects on the fish proving its role in improving the cytokine gene expression. Additionally, it exerted anti-parasitic potential against gill monogenean in *vitro*, which was supported in vivo. GE showed somewhat better results than OE. Further work is needed to investigate the other beneficial actions of GE and OE in various fish species for the sustainable aquaculture industry.

## Data Availability

All data supporting the findings of this study are available within the paper and its Supplementary Information.
